# Environmental Status and Geomorphological Characterisation of Seven Black Coral Forests on the Sardinian Continental Shelf (NW Mediterranean Sea)

**DOI:** 10.3390/biology11050732

**Published:** 2022-05-11

**Authors:** Davide Moccia, Laura Carugati, Maria Cristina Follesa, Rita Cannas, Pierluigi Carbonara, Antonio Pusceddu, Alessandro Cau

**Affiliations:** 1 Department of Life and Environmental Sciences, University of Cagliari, Via T. Fiorelli 1, 09126 Cagliari, Italy; laura.carugati@unica.it (L.C.); follesac@unica.it (M.C.F.); rcannas@unica.it (R.C.); apusceddu@unica.it (A.P.); alessandrocau@unica.it (A.C.); 2 COISPA Tecnologia & Ricerca, 70126 Bari, Italy; carbonara@coispa.it

**Keywords:** marine animal forest, environmental status, anthozoans, ROV, MSFD, DPSIR

## Abstract

**Simple Summary:**

Black coral forests are three-dimensional components of the marine mesophotic benthic community that play a crucial role in the benthic–pelagic processes, enhancing substrate complexity and creating numerous ecological niches and biodiversity hotspots. The increase of natural and human pressures on these forests is decimating their sophisticated architecture, leading to habitat degradation and biodiversity loss. This study assessed the environmental status of seven black coral forests dwelling in the centre of the Mediterranean Sea using the Mesophotic Assemblages Conservation Status Index. Our results showed how site-specific ecological conditions associated with different geomorphological settings can determine the variability of the environmental status among these habitats. Overall, most of the black coral forests investigated showed a “high” and “good” status; however, in two sites, a degraded benthic community and a marked anthropogenic impact determined a “moderate” and “poor” environmental status, highlighting the fragility of these communities to anthropogenic stressors, even in an area of low urbanisation, such as a Sardinian island. The scenario obtained by this study, combined with a more complete understanding of the processes that drive benthic communities’ dynamics, would facilitate the evaluation of potential measures for the appropriate management of human activities and the general conservation of mesophotic coral forests.

**Abstract:**

Marine animal forests are key mesophotic ecosystems that are under threat from increasing natural and human pressures. Despite the fact that various international agreements strive to preserve these fragile ecosystems, the environmental status of the majority of these animal-structured environments is unknown. Assessing their environmental status is the first step needed to monitor these essential habitats’ health over time and include them within conservation and protection frameworks, such as the Marine Strategy Framework Directive. Based on Multibeam data and ROV footage, we characterized the geomorphological setting and evaluated the environmental status of seven black coral forests in the centre of the Western Mediterranean Sea, using the Mesophotic Assemblages Conservation Status (MACS) Index. The presence of two antipatharians, *Antipathella subpinnata* and *Leiopathes glaberrima*, characterized the seven investigated sites, dwelling on rocky substrate characterized by different environmental drivers (i.e., depth, slope of the substrate, terrain ruggedness, topographic positioning index, and aspect). From the combined evaluation of the associated benthic community status and the anthropogenic impacts affecting it, a “high” and “good” environmental status was assessed for five out of the seven studied black forests, with only two forests classified as having a “moderate” and “poor” status, respectively. Overall, our study showed a site-specific variability of mesophotic black coral forest status, explained by different biological community structures and environmental conditions mainly associated with morphological and anthropogenic factors.

## 1. Introduction

Technological advancements have expanded the number of scientific studies of Mediterranean mesophotic ecosystems in recent decades, revealing the presence of extensive and diversified animal forests dwelling along most of the basin’s continental shelf [1,2]. Underwater photography and videography, integrated with seafloor imagery systems such as multibeam echo sounders (MBES), are now recognised as useful tools to shed light on the role of geomorphological and environmental factors shaping the distribution and characteristics of mesophotic coral reefs [3,4,5,6].

Antipatharians, generally known as “black corals” due to their chitinous black skeletons, are among the principal ecosystem engineers forming these benthic communities, as they develop three-dimensional frameworks that enhance the seabed’s complexity, creating numerous ecological niches and biodiversity hotspots [7]. Black coral forests occur at depths between 40 and 2048 m, from the Pacific Ocean to the northeast Atlantic Ocean, and between 60 and 600 m in the Mediterranean Sea [8,9]. Within the Mediterranean Sea, black coral forests have been found in the Sicilian and Sardinian Channels, as well as in the South Adriatic Sea, and in the Aegean Sea, often associated with different assemblages of hard-bottom-structuring species, such as the gorgonians *Eunicella cavolini*, *Callogorgia verticillata*, and *Paramuricea clavata* and scleractinians *Madrepora oculata*, *Lophelia pertusa*, and *Dendrophyllia cornigera* [10,11,12,13]. The main factors that modulate the distribution and aggregation patterns of these large anthozoans include their distinctive biological traits, such as slow development, late maturity, and short larval dispersal [14,15,16,17]. Additionally, environmental features such as depth, water current, availability of bedrock, slope inclination, and substrate complexity can influence their settlement success and, therefore, their species composition and spatial distribution [18,19,20].

Arborescent black corals offer shelter and feeding grounds for numerous associated species and play a crucial ecological role in the flow of energy from the pelagic to the benthic system [21,22]. In addition, they can represent crucial habitats for breeding and spawning through the ontogeny of several organisms [23]. On the other hand, due to their branching morphology and erect posture, black coral forests are also highly vulnerable to anthropogenic pressures (e.g., fishing activities, marine pollution, oil and gas exploitation), that can cause severe impacts to coral colonies, altering their ability to provide biological services, and therefore leading to the degradation of the habitats and the loss of biodiversity [1,24]. Fishing practices, such as bottom trawling, can cause the direct destruction of coral communities, while others (e.g., trammel net or longline) can cause abrasions and damage to colonies’ branches [25], which ultimately can lead to bacterial infections and epibiont colonisation [24]. Moreover, sediment resuspension and accumulation, often caused by trawling activities, gas and mineral inspections, global warming, and ocean acidification, are other potential threats to the environmental integrity of these habitats [25]. For these reasons, some black coral species, and the structural complexity that they provide, are regarded as vulnerable marine ecosystems (VMEs), encompassed by Annex II of the Barcelona Convention, and listed as “Near Threatened” in the International Union for Conservation of Nature’s (IUCN) Red List within the Mediterranean regional assessment, with *Leiopathes glaberrima* listed as “Endangered” due to its millennial life span [26]. Furthermore, they are listed as “declining species” by the OSPAR Commission (OSPAR Recommendation, August 2010) [27], and have been identified as special ecological features that require protection under the Convention of Biological Diversity [28]. Finally, the General Fisheries Commission for the Mediterranean (GFCM), as a Regional Fisheries Management Organization (RFMO) of FAO, has developed several recommendations (i.e., GFCM Recommendation 29/20) and regulations (i.e., the institution of Fisheries Restricted Areas, where the use of towed dredges and trawl-net fisheries at depths beyond 1000 m is prohibited) to protect these sensitive habitats [29,30].

Despite the ecological relevance (e.g., associated biodiversity, shelter effect, and energy flow) and vulnerability of these habitat-forming species, quantitative data on their distribution are still scarce, and the evaluation of their conservation status is not an easy task to achieve. In the Mediterranean Sea, a semi-enclosed and highly anthropised basin, pristine coral assemblages are constantly disappearing [31]; thus, assessing their environmental status, with a standardised and homogeneous method across the basin, is crucial for evaluating and monitoring their health over time, and possibly undertaking specific conservation actions.

The most relevant European environmental policy for the marine environment, the Marine Strategy Framework Directive (MSFD), requires the Member States to implement an ecosystem-based approach to reach or maintain a “Good Environmental Status” (GES) for marine habitats [32]. According to the MSFD, the GES of benthic habitats is evaluated with the use of three ecological descriptors: D1 (Biological diversity), D6 (Seafloor integrity), and D10 (litter). Accordingly, the Mesophotic Assemblages Conservation Status (MACS) Index assesses the environmental status of benthic assemblages, combining two independent indices: the Index of Status (I_s_), focused on biocoenotic complexity, and the Index of Impact (I_i_), focused on the anthropogenic impacts affecting the benthic community [33]. Recently, numerous methods have been used to assess the ecological quality of marine habitats (e.g., coralligenous reefs), such as the Ecological Status of Coralligenous Assemblages (ESCA) [34], the Coralligenous Bioconstructions Quality Index (CBQI) [35], the Coralligenous Assemblage Index (CAI) [36], the Coralligenous Assessment by Reef Scape Estimate (COARSE) [37], and the Mesophotic Assemblages Ecological Status (MAES) [38,39]. Compared to all these indices, the MACS Index focuses on mesophotic coral assemblages and follows the DPSIR (Driving forces—Pressures—Status—Impacts—Response) approach [40] derived from the MSFD protocol [32], and thus has the potential to be extended from a local, to a national, to even a European scale.

In this context, this study aims to describe the geomorphological setting and assess the environmental status of seven black coral forests dwelling along the Sardinian continental shelf. The acquisition of this knowledge is the first step in gathering the essential information needed to monitor their health and exposure to impacts over time and improving current frameworks for the conservation and protection of these crucial habitats.

## 2. Materials and Methods

### 2.1. Study Area

**Figure 1:**
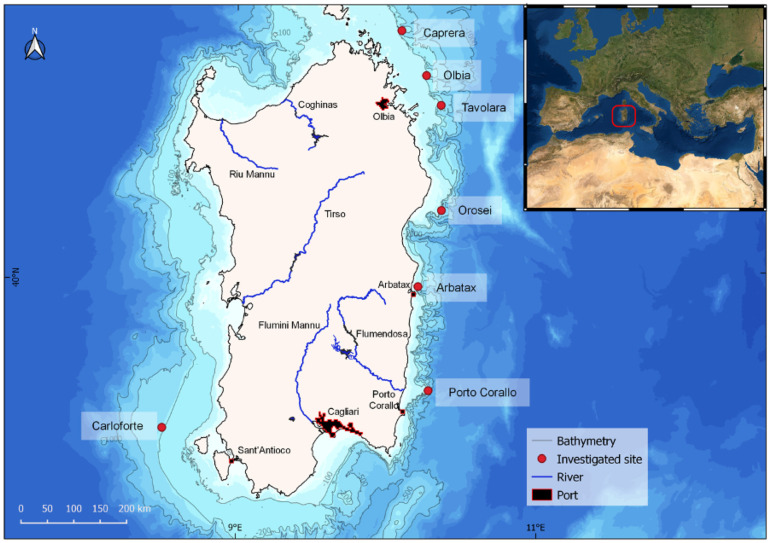
Map and isobaths of the Sardinian continental margin (West-Central Mediterranean Sea) with location of the study areas, main drainage network on land, and main Sardinian ports.

This study focused on seven mesophotic rocky reefs dominated by the presence of two black corals, *Antipathella subpinnata* and *Leiopathes glaberrima*, located at depths ranging from 82 m to 201 m along the continental shelf of the Island of Sardinia (in the Northwestern Mediterranean Sea) (Table S1; Figure 1). Five investigated sites (Caprera, Olbia, Orosei, Tavolara, and Arbatax) are located on the island’s northeastern coast, while one site (Porto Corallo) is located on the southeastern one (Figure 1). The eastern coast of Sardinia is characterized by a continental shelf with a narrow extension and a steep slope, incised by numerous submarine canyons [41]. The seventh site, known as Carloforte Shoal (hereafter Carloforte) [19], is located off the coast of the southwestern sector of the island, and it is characterised by the presence of a wide continental shelf, showing extensive areas of outcropping and suboutcropping of rocky volcanic substrates [42] (Figure 1). Anthropogenic pressures, mainly related to fishing activities and litter disposal along the Sardinian continental shelf, especially within submarine canyons, have been reported in numerous studies [43,44,45]. Six sites are located on the heads of submarine canyons, and only one is located around an isolated rocky outcrop within an extended continental shelf. These distinct geomorphological settings reflect the variability of the topographic conditions occurring along the Sardinian continental shelf. For this reason, we combine the analysis of high-resolution morpho-acoustic (Multibeam) data and ROV footage to evaluate how the different geomorphological and environmental conditions may affect the variability and the environmental status of Sardinian black coral forests and their associated communities.

### 2.2. ROV Surveys—Data Acquisition

This study did not involve damage to any species as the sampling was based on a non-invasive approach, with direct observations obtained by ROV footage and image analysis. The seven sites were investigated during two oceanographic surveys aimed at studying the status of *Corallium rubrum* populations along the Sardinian continental shelf carried out in October 2011 and July 2013 on board the *R*/*V Astrea*. Sites were defined as suitable for the present study only when, at least in 1 transect within the site, the presence of Antipatharians with density values higher than 0.1 colonies m^−2^ was observed. The *ROV ‘Pollux III’* was used to capture the footages, being equipped with a digital camera (Nikon D80, 10 megapixels (Tokyo, Japan)), a strobe (Nikon SB 400), and a high-definition video camera (Sony HDRHC7, Nihonbashi, Tokyo), 2 dimmable LED lamps (20 W max each, 3200 Lumen), a depth sensor, a compass, and an underwater acoustic positioning system. A total of 2 parallel laser beams were also installed aboard the ROV, giving a continuous 11 cm reference scale throughout the video frame. At each site, 3 video transects >200 m long (in accordance with the Italian MSFD protocol) were analysed, obtaining a total of 21 video transects from 13 ROV paths (Table S1). QGIS software was used to select the 200 m transects along the total tracks, and Apple Final Cut software was used to extrapolate the video footage with a 50 cm-wide visual field to cover at least 100 m^2^ of the bottom surface. Visual census of megabenthic species was carried out along the full extent of each transect. The area of the hard bottom (rocky or biogenic reefs), structuring species richness, density and heights, and the percentage of colonies exhibiting signs of epibiosis, necrosis, or direct entanglement in lost fishing gear were all documented using the MACS methodological procedure [33]. Marine litter was identified and counted, and the final density was computed considering the entire transect. Within each transect, 20 random, high-definition images targeting the hard bottom were obtained and used to calculate the basal living cover, coralline algae cover, and sedimentation level using CPCe (coral point count) software [46]. The modal value of each parameter was then calculated for each transect.

### 2.3. Multibeam Bathymetry

High-resolution morpho-bathymetric maps were obtained using a hull-mounted Multibeam echosounder (MBES; EM 2040 Kongsberg, 300 kHz frequency). Data were acquired with 40% lateral overlap and processed to remove spikes due to navigation and acquisition system problems. Data acquisition and processing were performed using the CARIS package (CARIS HIPS and SIPS 8.1.2, Fredericton, NB, Canada). The digital elevation models (DEMs) were obtained using a grid with a 5 × 5 m cell size. The software QGIS (Version 3.16.16, Hannover, Germany) was used to extract from the DEMs the data needed to characterise the ROV transects in regard to: (i) the slope of the sea bottom; (ii) the terrain ruggedness index (TRI), which provide an objective measure of habitat heterogeneity (as it is considered as an essential factor controlling species distribution [19,40,47]); (iii) the topographic positioning index (hereafter TPI), that defines the variation of the sea-bottom elevation with reference to the overall landscape, highlighting geomorphological features, such as depressions, outcrops, and flat areas; and (iv) the aspect, which provides information on the orientation of the seabed and, in turn, its potential exposure to prevailing currents [48] (Table 1; Figure S1).

### 2.4. Index Metrics

The MACS Index comprises two second-order independent indices, the Index of Status (I_s_) and the Index of Impact (I_i_), cumulatively composed of six metrics.

The I_s_ assesses the status of the benthic communities and is calculated throughout the analysis of: (i) the number of conspicuous benthic species (species richness—SR), taking into account only megabenthic sessile and sedentary hard-bottom species found in the intermediate and canopy layers; (ii) the living basal layer cover (BC), calculated as the percentage of the hard bottom covered by organisms that live in the basal (encrusting species) and intermediate layers (erect species less than 10 cm in height) and classified as follows: 0, <30%, 30–60%, >60%; (iii) coralline algae cover (CC), calculated as the percentage of basal living cover represented by encrusting coralline algae and categorized as: none, sparse, abundant, or highly abundant; (iv) the canopy condition, based on the number of dominant structuring species that construct the canopy (DM); (v) the density of all structuring species (SSD), expressed as the number of colonies or individuals m^−2^ ± SE; and (vi) the mean height of the dominant structuring species (SSH), described in cm ± SE. A score from 0 to 3 was assigned for each metric, considering 3 as the maximum value when the ‘ideal’ reference condition, in terms of biodiversity, living cover in the basal layer, and canopy development, was observed across the transect. (See reference value in Table S2.)

The I_i_ assesses the impact status and is calculated throughout the analysis of: (i) the percentage of hard bottoms covered by sediment (SD) and classified as: 0%, <30%, 30–60%, or >60%; (ii) the percentage of colonies directly entangled with marine litter or fishing gear (ENT); (iii) the percentage of colonies showing necrotic portions (NCR); (iv) the percentage of colonies showing portions with epibionts (EPB); (v) the density of marine litter (LD) expressed as number of items ± SE; and (vi) the type of litter (LT) distinguishing in: general debris (low impact), lost fishing gear (high impact), or both (maximum impact). As for the I_s_, a score from 0 to 3 was assigned for each I_i_ metric, considering 0 as the ‘ideal’ reference condition, assigned in the case of the absence of silt cover, damaged structuring anthozoans, and marine litter.

To obtain the ecological quality ratio (EQR), the I_s_ and I_i_ metrics were normalised by dividing by 3 (the maximum value expected) and then multiplying by 100 (the scale of the EQR) (see Supplementary Material File S2). After transforming the scores of the 12 metrics in the EQR, the 2 indices were calculated for each transect by summing all of the EQR values (6 per index) and then dividing by 6:

(EQR_SR_ + EQR_BC_ + EQR_CC_ + EQR_DM_ + EQR_SSD_ + EQR_SSH_)/6 = I_s_ (1)

(EQR_SD_ + EQR_ENT_ + EQR_NCR_ + EQR_EPB_ + EQR_LD_ + EQR_LT_)/6 = I_i_ (2)

The final MACS Index is a third-order index that combines information from both I_s_ and I_i_ and is calculated for each site using the following formula:

(I_s_ + (100 + I_i_))/2 = MACS (3)

I_s_, I_i_, and the resulting MACS Index give a value range from 0 to 100, referenced to 5 classes, identified following the water framework directive classification. (See Table S2 in Supplementary Material File S1.)

### 2.5. Statistical Analyses

We performed a canonical correspondence analysis (CCA) [45] to illustrate the potential relationship between the 7 black coral forest sites, characterised by the 12 metrics used to assess their environmental status, and the geomorphological variables (depth, slope, TRI, and TPI). CCA is a constrained ordination method, where axes are created through linear environmental variable combinations to detect which one ‘best’ describes a variation [49]. CCA analysis was carried out using routines included in the PAST program [50]. The statistical significance of the contribution of each variable to each CCA axis was tested using permutational simulation. The length of the arrows (geomorphological parameters) and orientation indicate their relative importance and approximate correlations to the axes.

## 3. Results

### 3.1. Geomorphological Setting

**Figure 2:**
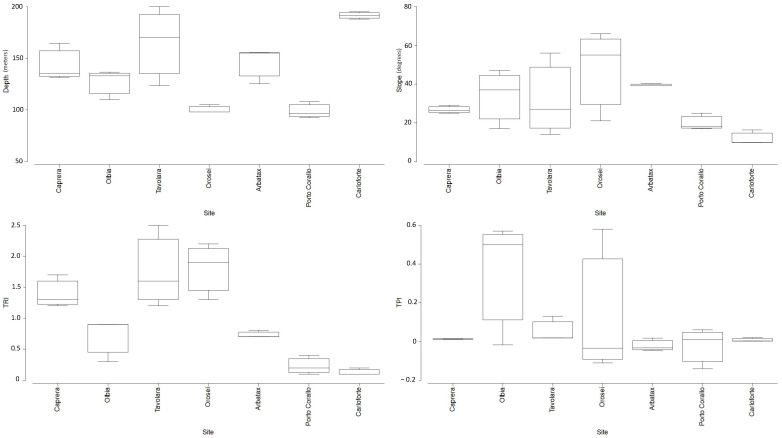
Boxplots showing the geomorphological characteristics of the investigated sites. TRI, terrain ruggedness index; TPI, topographic positioning index.

The geographic and geomorphological features of the 7 investigated sites are summarised in Table S1 and represented in Figure 2. The bathymetric range of the investigated black coral forests varied from 99 m in Porto Corallo to 191 m in Tavolara.

The steeper slope was recorded in transects located on the head of submarine canyons, with the highest values found in Orosei (Ave. of 47° ± 13.5 SE), followed by Arbatax, Olbia, and Tavolara (Ave. of 39° ± 0.3 SE, 34° ± 8.8 SE, and 32° ± 12.4 SE, respectively). A gentler slope was recorded in Caprera and Porto Corallo (Ave. of 27° ± 1.1 SE and 20° ± 2.5 SE, respectively), while the lowest slope was found in Carloforte (Ave. of 12° ± 2.0 SE).

The transects in Olbia showed the highest sea bottom elevation (TPI average of 0.35 ± 0.18 SE), followed by Orosei (0.14 ± 0.21 SE), Tavolara (0.05 ± 0.03 SE), Caprera (0.01 ± 0.001 SE), Arbatax (0.01 ± 0.02 SE), and Carloforte (0.008 ± 0.006 SE). Transects in Porto Corallo were characterized by a more marked sea-bottom depression (average TPI −0.023 ± 0.06 SE).

The highest topographic complexity was observed in Orosei (TRI average of 1.83 ± 0.25 SE), followed by Tavolara (1.75 ± 0.39 SE), Caprera (1.39 ± 0.13 SE), Arbatax (0.72 ± 0.02 SE), and Olbia (0.69 ± 0.2 SE). The lowest TRI values occurred in Porto Corallo (0.25 ± 0.1 SE) and Carloforte (0.17 ± 0.03 SE) (Table S1).

The sea bottom along the Tavolara and Olbia transects was mainly oriented to the east, while transects in Porto Corallo and Orosei were mostly westerly oriented. Caprera and Carloforte showed a dominant southerly oriented aspect, whereas transects in the Arbatax site were mainly oriented to the north (Table S1).

#### Coral Assemblages and Anthropogenic Impacts

A total of 86 species belonging to 9 *phyla* (Porifera, Cnidaria, Echinodermata, Bryozoa, Brachiopoda, Annelida, Mollusca, Arthropoda, and Chordata) were recognised across all studied areas (Table S3). Conspicuous megabenthic species varied among transects, from an average of 19 species (15–24) in Arbatax to an average of 39 (34–45) in Tavolara.

The highest basal living cover was found in Tavolara and Caprera (average of 62% ± 28 SE and 44% ± 15 SE, respectively), where encrusting polychaetes and sponges were the dominant taxa, followed by Carloforte (30% ± 5 SE), Olbia (27% ± 11 SE), and Orosei (25% ± 7 SE). The lowest basal living cover occurred in Arbatax (12% ± 4 SE). The coralline algae cover was abundant in Orosei, sparse in Caprera and Porto Corallo, and utterly absent in Olbia, Tavolara, and Carloforte.

A total of 17 structuring (forming the canopy) species (with over 5342 individuals and colonies counted) was recorded along all the video-transects: 3 sponges (*Axinella* spp., *Pachastrella monilifera*, and *Poecillastra compressa*), 3 scleractinians (*Dendrophyllia cornigera*, *Desmophyllum dianthus*, and *Madrepora oculata*), 4 antipatharians (*Antipathies dichotoma*, *Antipathella subpinnata*, *Paranthipates larix*, and *Leiopathes glaberrima*), 6 gorgonians (*Corallium rubrum*, *Paramuricea clavata*, *Acanthogorgia hirsuta*, *Eunicella cavolini*, *Viminella flagellum*, and *Callogorgia verticillata*), and 2 bryozoans (*Myriapora truncata*, *Reteporella beaniana*).

Dense forests of *L. glaberrima* dominated the sites in Carloforte and Tavolara, with a mean density of 0.8 ± 0.1 and 1.9 ± 0.1 col m^−2^ and an average height of 58 ± 1.6 and 55.2 ± 1.3 cm, respectively (Figure S2). *A. subpinnata* colonies dominated Porto Corallo, Orosei, Caprera, Olbia, and Arbatax transects with a mean density of 0.8 ± 0.008, 1.1 ± 0.3, 0.5 ± 0.1, 0.3 ± 0.1, and 0.1 ± 0.04 col m^−2^, respectively, and an average height of 54.2 ± 1.1, 36.9 ± 4.3, 34.8 ± 2.5, 15.3 ± 1.3, and 36.4 ± 6.3 cm (Figure S2).

Canopies were generally multispecific, with forests of *A. subpinnata* associated mostly with *C. rubrum*, *E. cavolini*, *C. verticillata*, and *V. flagellum* colonies, and forests of *L. glaberrima* associated mostly with *Axinella* spp., *P. monilifera*, *P. larix*, and *E. cavolini* (Figure S2). Overall, the highest densities of structuring species were found along transects in Tavolara (2.5–5.2 col m^−2^) and Orosei (1.7–4.7 col m^−2^). The lowest densities of structuring species occurred in Arbatax (1.2–2.1 col m^−2^).

The percentage of hard bottom covered by sediment was high in Carloforte (ca. 89% ± 4 SE), intermediate in Olbia and Arbatax (45% ± 11 SE and 44% ± 12 SE, respectively), and low in Tavolara, Orosei, and Caprera (33% ± 7 SE, and 22% ± 2 SE, respectively). In Porto Corallo, the SD score showed a value of 0 due to the low percentage of bottom cover and to the presence of the mouth of the river basin of the Flumendosa within 60 km of the investigated site, which, by protocol, further decreases the SD values. (For more details, see [33].)

The highest percentage of anthozoan colonies entangled in fishing gear was found in Orosei (7% ± 0.8 SE), followed by Tavolara (4% ± 0.2 SE). All the other sites showed a percentage of entangled colonies < 2%, except for Porto Corallo and Carloforte, in which <1% of the colonies were affected.

The highest percentage of necrotic colonies was observed in Arbatax (Ave. 27% ± 3.4 SE), followed by Olbia and Caprera (14% ± 1.9 SE and 9% ± 2.1 SE, respectively), Orosei (4% ± 1.1 SE), Tavolara (3% ± 0.8 SE), Porto Corallo and Carloforte (each with 1% ± 0.1 SE).

The highest percentages of epibiont-covered colonies were found in Caprera and Arbatax (Ave. 13% ± 2.7 SE and 11% ± 1.4 SE, respectively), whereas in all other sites, except for Porto Corallo (2% ± 0.6 SE), the percentage of epibiont-covered colonies was 5% ± 3.4 SE.

**Figure 3:**
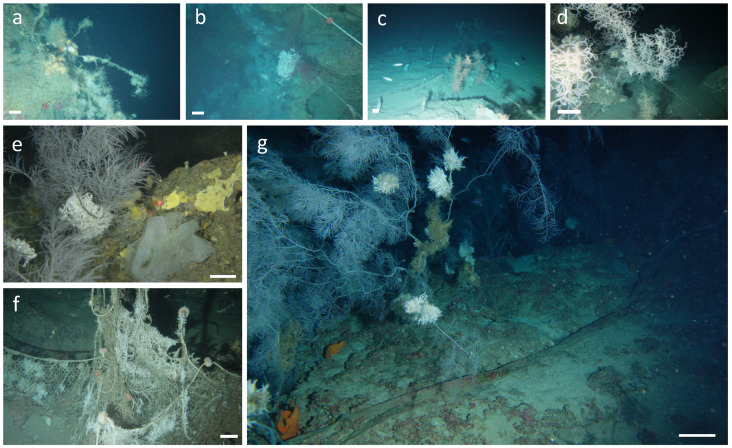
ROV images of anthropogenic impacts on the study sites. (**a**) Necrotic and epibiont-covered colonies of *A. subpinnata*; (**b**) big trammel nets entangled on *A. subpinnata* colonies; (**c**,**d**) fishing-lines entangled on *L. glaberrima* colonies; (**e**) plastic bag next to a *A. subpinnata* colony; (**f**) epibiont-covered fishing net; (**g**) long, undefined object lying next to a tall *A. subpinnata* colony. Scale bar of approximately 10 cm.

Marine litter was found in all sites (Figure 3), with densities ranging from 0.001 ± 0.001 SE items 100 m^−2^ in Carloforte transects, to 0.08 ± 0.04 SE items 100 m^−2^ in Arbatax. Lost fishing gear (mainly longlines, nets, and ropes) were found in all sites, whereas other litter categories occurred only in Porto Corallo, Caprera, and Arbatax.

### 3.2. Index Outcomes

**Figure 4:**
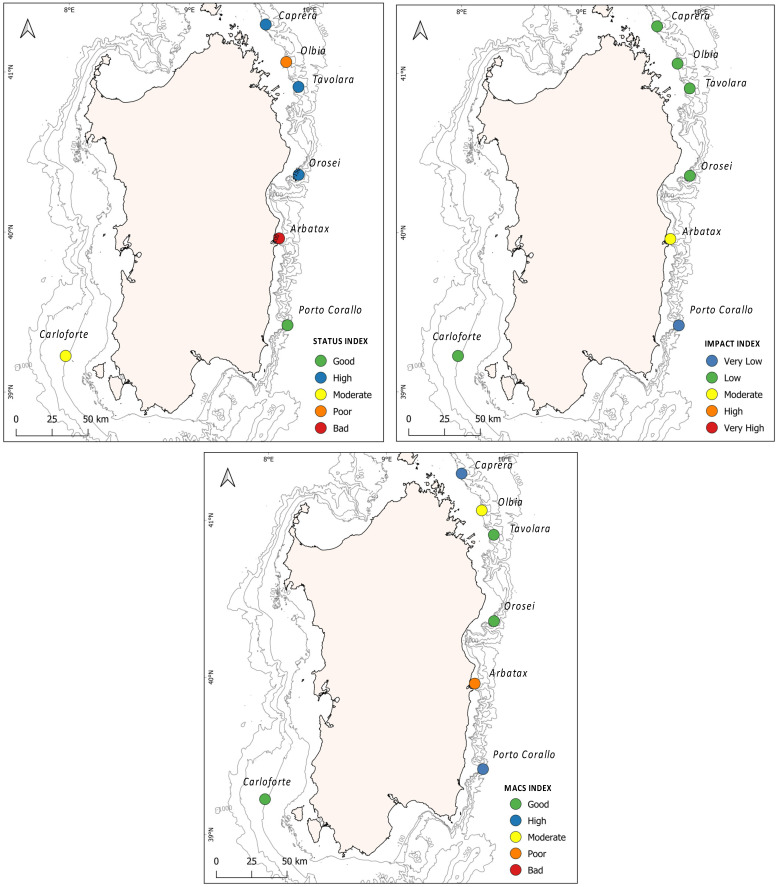
Results of the Index of Status (I_s_), Index of Impact (I_i_), and MACS Index for the seven investigated sites. Refer to Table S1 and Table 1 for site identifications and index results.

The 7 studied sites showed values of the MACS Index comprised between 38 and 68, with the I_s_ ranging from 24 to 74, and the I_i_ from 22 to 48 (Table 1; Figure 4).

I_s_ scores reflecting a “high” status occurred in Caprera, Tavolara, and Orosei (I_s_ = 74, 69, and 69, respectively), whereas the status was “good” in Porto Corallo (I_s_ = 56), “moderate” in Olbia (I_s_ = 54), and “poor” in Carloforte (I_s_ = 41). A “bad” status was recorded only in Arbatax (I_s_ = 24) (Table 1; Figure 4).

The highest impact was recorded in Arbatax (I_i_ = 48), whereas lower impacts occurred in Carloforte, Caprera, Tavolara, Olbia, and Orosei (I_i_ = 41,40, 39, 39, and 38 respectively). The least impacted site was Porto Corallo (I_i_ = 22) (Table 1; Figure 4).

Overall, the environmental status of the investigated black coral forest was “high” in Caprera and Porto Corallo (MACS = 68 and 67, respectively). Orosei, Tavolara, and Carloforte showed a “good” environmental status (MACS = 65, 64, and 57, respectively), Olbia and Arbatax showed a “moderate” status (MACS = 46) and a “poor” status (MACS = 37), respectively (Table 1; Figure 4).

**Figure 5:**
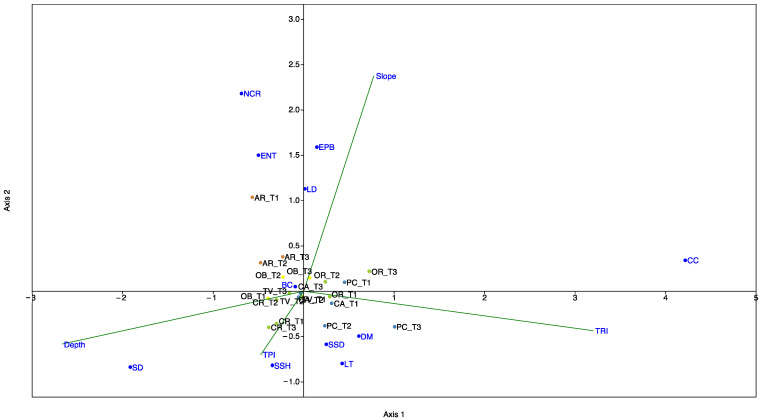
Outputs of the canonical correspondence analysis (CCA) performed using environmental variables (depth, slope, TRI, and TPI) and the 12 metrics used to assess the environmental status of the 7 sites (blue dots). SR: species richness; BC: living basal layer cover; CC: coralline algae cover; DM: dominant structuring species; SSD: density of all structuring species; SSH: mean height of the dominant structuring species; SD: sediment, ENT: percentage of colonies directly entangled in marine litter or fishing gear; NCR: percentage of colonies showing necrotic portions; EPB: percentage of colonies showing parts with epibionts; LD: density of marine litter LT: type of litter. Sites are labelled with site_transect number and coloured according to their environmental status.

The relationship between the seven investigated forests characterized by their environmental status and geomorphological variables (depth, slope, TPI, and TRI) are presented in the CCA triplot (Figure 5). The first two axes accounted for 93% of the total variance (axis 1 = 63%; axis 2 = 30%), and the permutation test was significant (eigenvalue for axis 1 = 0.07 and for axis 2 = 0.04, *p* < 0.05). The CCA plot showed that axis 1 was positively correlated to the TRI and negatively correlated to depth. Axis 2 was positively correlated with slope and negatively correlated to TPI. This indicated an increase in TRI, together with a decrease in depth from the left to the right of the CCA ordination diagram, and an increase in slope with a decrease in TPI from the bottom to the top of the CCA ordination diagram. Therefore, the CCA diagram shows how the sites with good and high environmental statuses (Porto Corallo, Orosei, Tavolara, and Carloforte) correlated to having high TRI values in both shallower and deeper depths, while those with poorer statuses were correlated to having low TRI and TPI scores and high depth and slope scores.

## 4. Discussion

Very few studies have focused on assessing the actual environmental status of mesophotic ecosystems and, thus, on collecting the quantitative information needed to determine the baseline for developing appropriate conservation policies and future monitoring activities. The increase of human impacts, mainly due to fishing pressures and uncontrolled litter disposal, is leading to the severe degradation of these important mesophotic ecosystems [1,51,52], stressing the need to enforce control and surveillance measures to improve and preserve the “Good Environmental Status” of mesophotic coral forests under European marine legislations. While shallower ecosystems, such as coastal mangroves and seagrasses, as well as deeper, cold-water coral reef habitats, are currently under several monitoring and protection measures for the maintenance and restoration of these ecosystems [53,54], only a few conservation initiatives are explicitly aimed at the protection of deep circalittoral assemblages from destructive practices, such as fishing activities [28,30]. To provide cues on these issues, in this study, by means of the MACS Index, we assessed the environmental status of seven black coral forests dwelling along the Sardinian continental shelf and related it to a set of geomorphological characteristics of the sea bottom.

Black coral forests represent an important component of the extensive mesophotic coral assemblages that occur along the continental shelf of Sardinia [55]. The presence of these animal-dominated habitats has been documented by numerous authors across the whole Mediterranean basin [56,57,58,59,60], underlining their importance as essential habitats, nursery grounds, and biodiversity hotspots [61,62,63,64]. In this context, our results expand the knowledge of black coral forests occurrence in the Italian and Mediterranean Seas [60], confirming a shallower bathymetric distribution for *A. subpinnata* (between 82 and 178 m) compared to the deeper one of *L. glaberrima* (between 180 and 200 m) [3,65]. *A. subpinnata* forests recorded along the transects in Caprera, Orosei, and Porto Corallo were documented dwelling with several other three-dimensional structuring species, such as the gorgonians *E. cavolinii* and *C. rubrum* and the porifera *Axinellae* spp., *P. compressa*, and *P. monilifera* (Figure S2). The association of *A. subpinnata* colonies with gorgonians and sponges has already been described by different authors, as they often prefer specific high-energy environments, characterised by low sedimentation rates and high food availability [57,61]. The Tavolara site was characterized by a dense and tall forest of *L. glaberrima*, accompanying numerous colonies of *P. larix*, *D. cornigera*, and *M. oculata* species mainly found in more profound and more oligotrophic waters [66,67]. The presence of these polyspecific coral assemblages creates favourable conditions for the development of a heterogeneous fauna living on the basal (e.g., encrusting sponges, bryozoans, and polychaetes) and intermediate (e.g., echinoderms and fish) canopy layers, showing a high and good status of the benthic communities (Table 1; Figure 4). This situation can be associated with geomorphological characteristics recorded in these sites, as they dwell on the head of submarine canyons characterised by rocky substrates with high topographic complexity (high values of TRI and TPI) (Figure 5). These results corroborate the relevant biological and ecological role on the abundance and diversity of benthic communities related to the elevated substrate heterogeneity described by several studies [68,69,70,71,72]. On the other hand, the lower geomorphological complexity documented along Carloforte transects, characterised by high sedimentation cover and low substrate availability, can explain the moderate value of the benthic community status found within the Carloforte black coral forest, where low species richness and low living basal cover levels were recorded (Table S1; Figure 4). Finally, the lowest benthic community status values were found in the Olbia and Arbatax forests, as they were characterised by low values of species richness for both basal living cover and associated structuring species (Table 1, Figure 4). Since both of these sites showed a high geomorphological complexity, we assume that the low I_s_ found could be related to their proximity to two important ports (<13 km Olbia port and <5 km from Arbatax port, Figure 1) and, in turn, to a potentially higher presence of marine traffic and fishing activities. Although rocky substrata are usually not trawled by fisherman, these activities are generally more intense in the proximity of canyon heads [73] and can produce a strong resuspension of fine sediment that increases water turbidity and could negatively affect benthic assemblages [74,75].

In general, a low anthropogenic impact was recorded among the investigated black forests, with the lowest I_i_ value documented along the transects carried out in Porto Corallo, showing a very low impact (Table 1; Figure 4). These results are consistent with other studies showing how Sardinian sea bottoms appear less impacted than other Mediterranean regions [43,44]. However, within all of the investigated forests, the presence of lost fishing gears found entangled on coral colonies and lying on the seafloor was evident (Figure 2). The highest value of I_i_ was found along the transects carried out in Arbatax, where a combination of a high percentage of sediment cover, necrotic colonies, and litter density was documented (Table 1; Figure 4; Supplementary Material File S2). As speculated for the benthic community status, the elevated impact recorded for this site could be linked to its vicinity to the port of Arbatax (<5 km, Figure 1), from which fishing vessels can easily and quickly reach the proximity of the investigated forest. The destructive effect of bottom trawling is well-documented on these assemblages [76,77]. However, the impact of smaller artisan fishing gear, such as trammel nets and longlines, can be severe as they cause abrasion of the colonies, possibly triggering bacterial infections and epibiont colonisation, leading to the necrosis of the entire colony [24]. Generic litter items were rarely found across all of the studied forests. This aspect may be linked to hydrodynamic and anthropogenic factors characterising the studied sites. In areas such as Tavolara, Orosei, and Arbatax, the flushing effect, typically existing on the head of submarine canyons systems, can convey litter objects to the bottom of the canyon axes [77], explaining the absence of waste disposal in these sites. On the other hand, the presence of general litter, mainly plastic bags, found within the Olbia forest may be linked to the important maritime traffic that connects the Olbia harbour to the Italian mainland [78].

The calculation of the MACS Index for the investigated black coral forests confirmed an overall good environmental status of Sardinian mesophotic assemblages [13,33,38], with only two sites that failed to reach this goal (Figure 4). The combination of a good and high benthic communities’ status accompanied by a low impact affecting them determined a high to good MACS scores for Porto Corallo, Caprera, Tavolara, Orosei and Carloforte forests (Table 1; Figure 4). The CCA diagram shows how in these sites the habitat characteristics that act on a small spatial scale, such as those derived from high geomorphological complexity, could represent important factors that favour the development of abundant and diverse benthic communities living in a healthy status (Figure 4). In addition, the lower impacts recorded across the Sardinian mesophotic assemblages compared to other Mediterranean areas [33,39,63], may be the consequences of the low degree of coastal urbanisation (67 people km^−2^, Italian National Statistical Institute—ISTAT, http://dati.istat.it, accessed on 10 September 2021), fishing pressure, and waste pollution along the Sardinian coasts. The lowest environmental status value was found in Arbatax, in which the higher anthropogenic impact feasibly affected the integrity of the benthic communities, resulting in an overall poor environmental status (Table 1; Figure 4). Several authors highlighted the correlation between the proximity and the intensity of coastal urbanisation and low environmental status for mesophotic assemblages and shallower coralligenous habitats [26,36,37,67].

## 5. Conclusions

The present study contributes to the knowledge on the distribution and environmental status of mesophotic black coral forests in the Mediterranean Sea. Our results confirm that environmental status assessment through the multiparametric MACS Index represents a valuable tool that allows for relatively fast and easy comparative evaluations of standardised data, even for hard-to-reach environments. Our results show how site-specific ecological conditions associated with different geomorphological settings and potential anthropogenic impacts could determine variability of the environmental status among these ecosystems. The heterogenic settings characterising the Sardinian continental shelf resulted in an overall high to good status ratings for most of the black coral forests investigated, emphasising the favourable ecological conditions occurring along the Island of Sardinia. However, a poor benthic community and a marked anthropogenic impact found in Olbia and Arbatax determined a moderate and poor environmental status, highlighting the fragility of these communities to anthropogenic stressors, even in a less urbanised area, such as the Island of Sardinia. The scenario obtained from this study, combined with a more complete understanding of the most relevant processes that drive benthic communities’ dynamics, related to geomorphological, oceanographic and biological characteristics (topographic complexity, pelagic larval dispersal and settlement factors, and population connectivity), would facilitate the evaluation of potential measures for the appropriate management of human activities, habitat recovery, and the general conservation of mesophotic coral forests. However, further applications of the MACS Index from other Mediterranean mesophotic coral habitats, characterised by different environmental and anthropogenic pressures, are needed so as to apply a larger scale to the monitoring of their health and impacts statuses over time. Under the European Marine Strategy Directive, such knowledge is fundamental in planning future potentially protected areas needed to achieve a “Good Environmental Status” for these valuable and vulnerable habitats.

## Figures and Tables

**Table 1:** Scores obtained for the 12 metrics, the Status Index, the Impact Index and the MACS Index in the 7 investigated sites.

	Status Index (I_s_)						Impact Index (I_i_)						I_s_	I_i_	MACS
Site	SR	BC	CC	DM	SSD	SSH	SD	ENT	NCR	EPB	LD	LT			
Caprera	67	78	33	100	67	100	33	11	33	44	33	78	74	39	68
Olbia	67	56	0	44	44	33	56	11	26	33	33	78	41	39	51
Tavolara	89	89	0	89	78	67	40	33	33	33	33	67	69	40	64
Orosei	56	56	100	78	55	67	29	33	22	44	33	67	69	38	65
Arbatax	22	44	0	11	22	45	44	11	89	33	44	67	24	48	38
Porto Corallo	67	44	67	56	56	44	0	11	11	11	22	78	56	22	67
Carloforte	67	67	0	45	44	100	89	22	11	22	22	78	54	41	57

(SR, species richness; BC, basal biocover; CC, coralline algae cover; DM, dominance; SSD, structuring species density; SSH, structuring species height; SD, sedimentation; ENT, entanglement; NCR, necrosis; EPB, epibiosis; LD, litter density; LT, litter type).

## Data Availability

Data are contained within the article or the Supplementary Materials.

## Supplementary Materials

The following supporting information can be downloaded at: https://www.mdpi.com/article/10.3390/biology11050732/s1, Supplementary Material File S1: Figure S1: Bathymetric, TRI (a), Slope (b), and Aspect (c) map of the study areas from MBES data, showing the ROV navigation tracks (red lines), the site IDs, and the approximate locations of the 200-m long (A, B, C) transects extracted for the quantitative analyses and application of MACS Index, Figure S2: Selected ROV images showing black coral colonies at the seven investigated sites: (a) Colonies of *A. subpinnata* observed at the Caprera site at 144 m depth with colonies of *E. cavolinii* and *P. compressa* underneath the canopy; (b) colonies of *A. subpinnata* at the Olbia site at 133 m depth, with a dense facies of *V. flagellum*; (c) colonies of *L. glaberrima* on an encrusted, rocky boulder at the Tavolara site at 177 m depth; (d) colonies of *A. subpinnata* at the Orosei site at 98 m depth, with several portions of the branches covered by *Salmicina-Filigrana implexa*; (e) a big colony of *A. subpinnata* at the Arbatax site at 140 m depth, with portions covered by the epibionts *Salmicina-Filigrana implexa* and other polychaete species; (f) a thick forest of *A. subpinnata* at the Porto Corallo site at 95 m depth, with a few individual specimens of *A. anthias* swimming underneath the canopy; (g) colonies of *L. glaberrima* at the Carloforte site at 184 m depth, with capsules of *S. caniucla* hanging over the branches. Scale bar of approximately 10 cm, Table S1: Geographic and geomorphological characteristics of ROV dives performed in the seven investigated sites. TRI: terrain ruggedness index, TPI: topographic positioning index, Table S2: (A) Reference scores developed for the 12 components used in the MACS Index. H, height; GL, general litter; LFG, lost fishing gear. (B) Scores defining the environmental status classes for I_s_, I_i_, and MACS. Both tables values are obtained from Enrichetti et al. (2019), Table S3: List of species recorded within the investigated sites, Supplementary Material File S2: Table with values of the 12 metrics and their conversion in EQR.
