# Supplementary material for: Environmental Status and Geomorphological Characterisation of Seven Black Coral Forests on the Sardinian Continental Shelf (NW Mediterranean Sea)
Source: Biology (Basel). 2022 May 11;11(5):732. doi: 10.3390/biology11050732 (PMC9138414; doi:10.3390/biology11050732)
Supplement: Supplementary file 1 [file biology-11-00732-s001.zip › Supplementary Material S1.pdf]

SUPPLEMENTARY MATERIAL S1

Environmental Status and Geomorphological Characterisation of Seven Black Corals Forests from Sardinian Continental Shelf (NW Mediterranean Sea)

Davide Moccia[1*], Laura Carugati[1], Maria Cristina Follesa[1], Rita Cannas[1], Pierluigi Carbonara[2], Antonio Pusceddu[1] and & Alessandro Cau[1]

[1]Department of Life and Environmental Sciences, University of Cagliari, Via T. Fiorelli 1, 09126 Cagliari, Italy.
[2]COISPA Tecnologia & Ricerca, Bari, Italy
* Correspondence: Davide Moccia: mocciadavide@unica.it; Postal address: Dipartimento delle Scienze della Vita e dell'Ambiente, Università di Cagliari, Via Fiorelli 1, Cagliari, 09126, Italy.

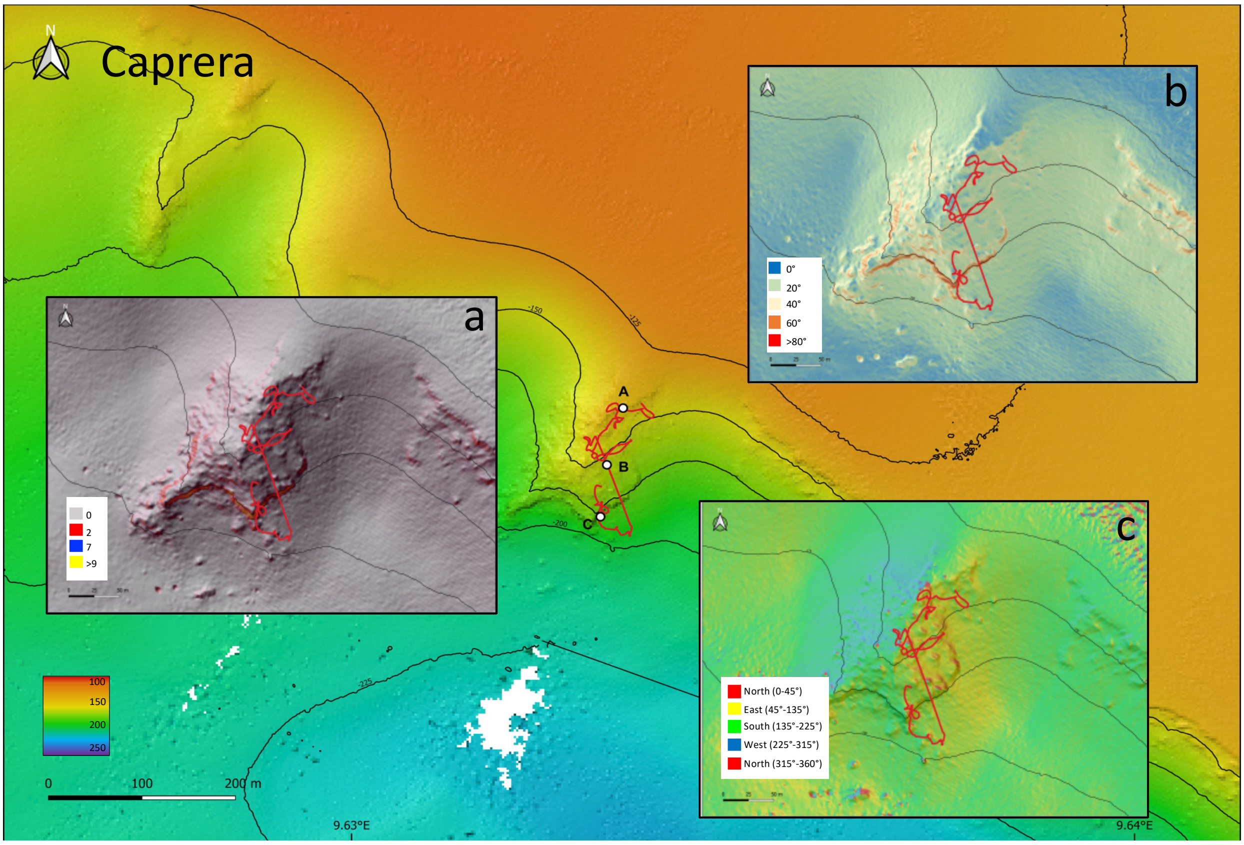
Caprera
a
b
c
A
B
C
0
2
7
>9
0°
20°
40°
60°
>80°
North (0-45°)
East (45°-135°)
South (135°-225°)
West (225°-315°)
North (315°-360°)
100
150
200
250
0
100
200 m
9.63°E
9.64°E

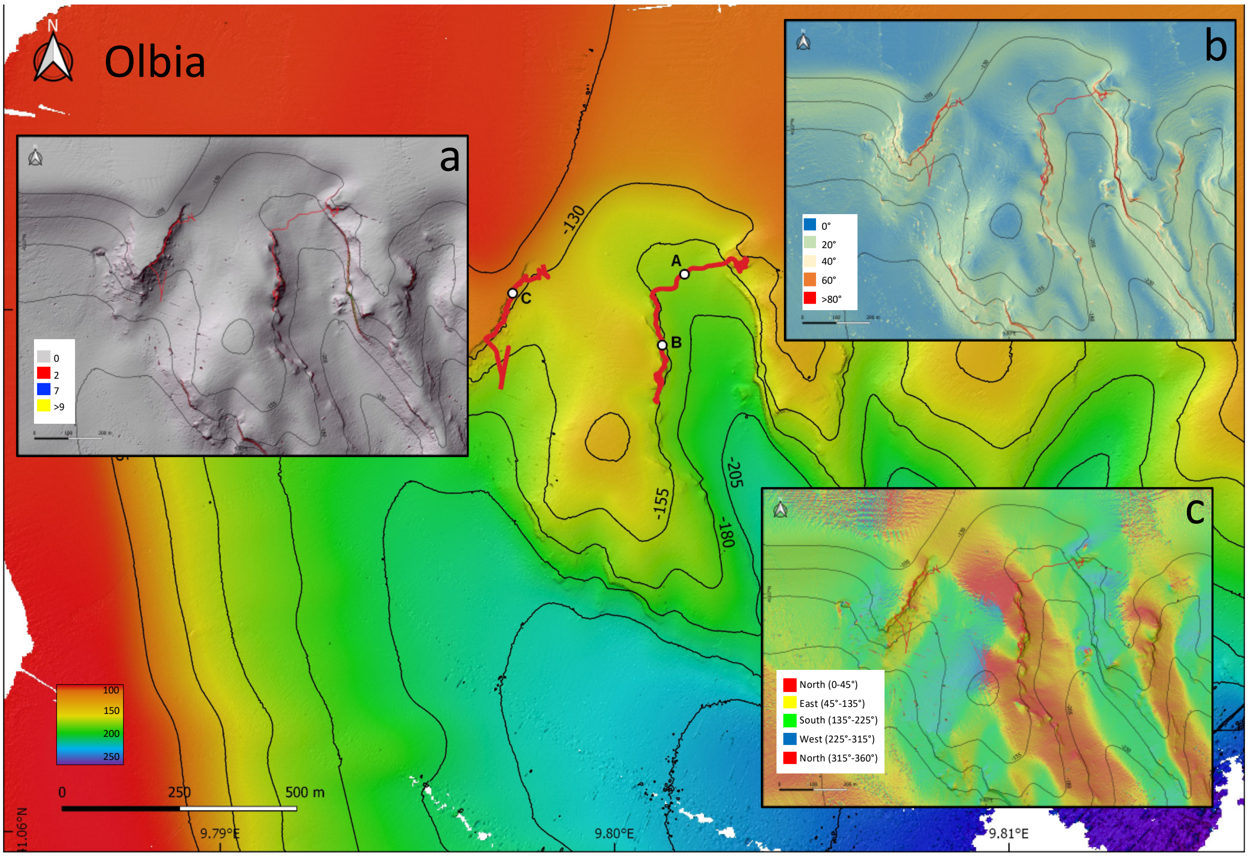
Olbia
a
b
c
A
B
C
-130
-155
-180
-205
0
2
7
>9
0°
20°
40°
60°
>80°
North (0-45°)
East (45°-135°)
South (135°-225°)
West (225°-315°)
North (315°-360°)
100
150
200
250
0
250
500 m
9.79°E
9.80°E
9.81°E

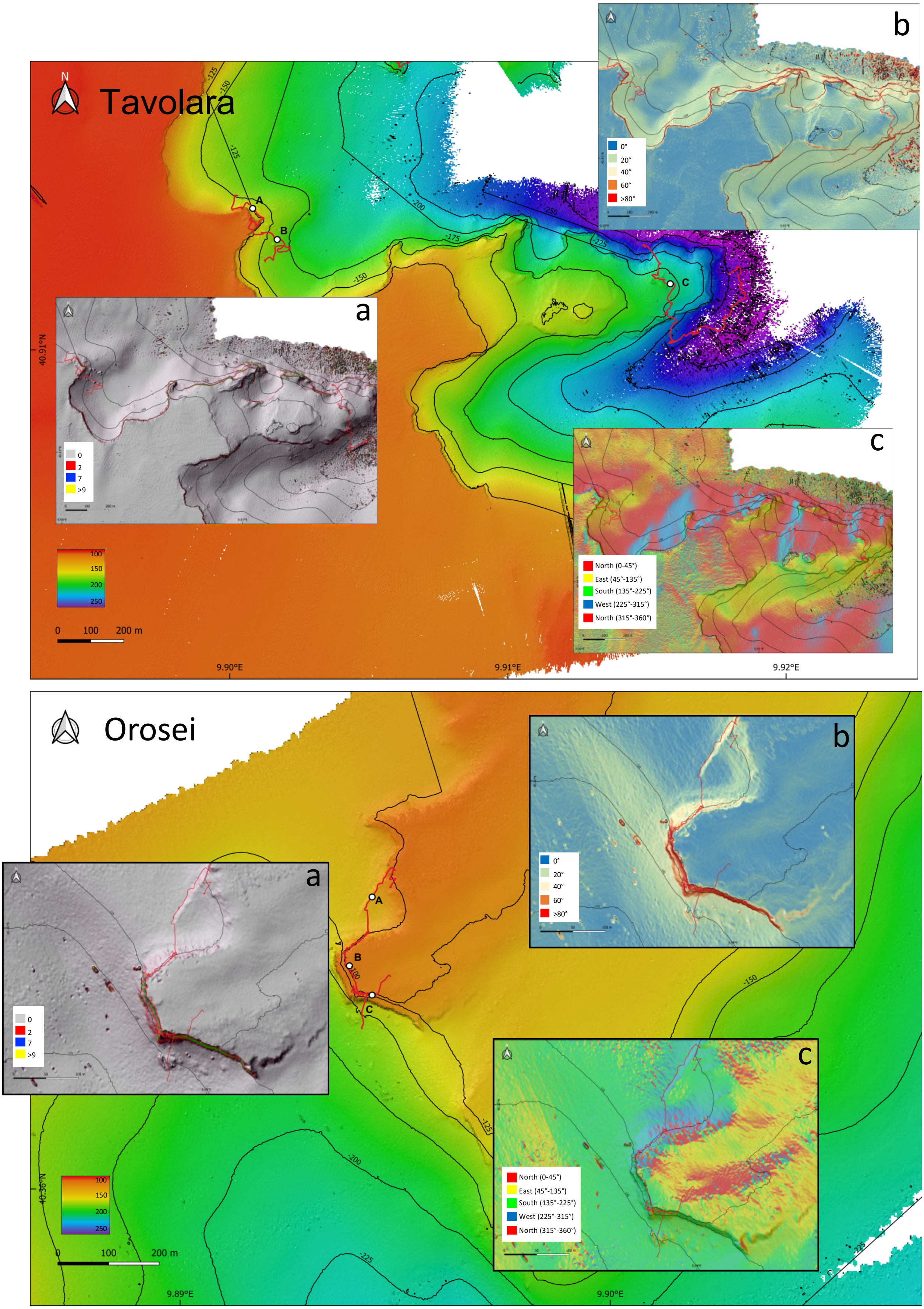
N
Tavolara
-125
-150
-175
-200
-225
-250
A
B
C
a
0
2
7
>9
b
0°
20°
40°
60°
>80°
c
North (0-45°)
East (45°-135°)
South (135°-225°)
West (225°-315°)
North (315°-360°)
100
150
200
250
0
100
200 m
40.91°N
9.90°E
9.91°E
9.92°E
Orosei
A
B
C
-100
-125
-150
-200
-225
a
0
2
7
>9
b
0°
20°
40°
60°
>80°
c
North (0-45°)
East (45°-135°)
South (135°-225°)
West (225°-315°)
North (315°-360°)
100
150
200
250
0
100
200 m
40.36°N
9.89°E
9.90°E

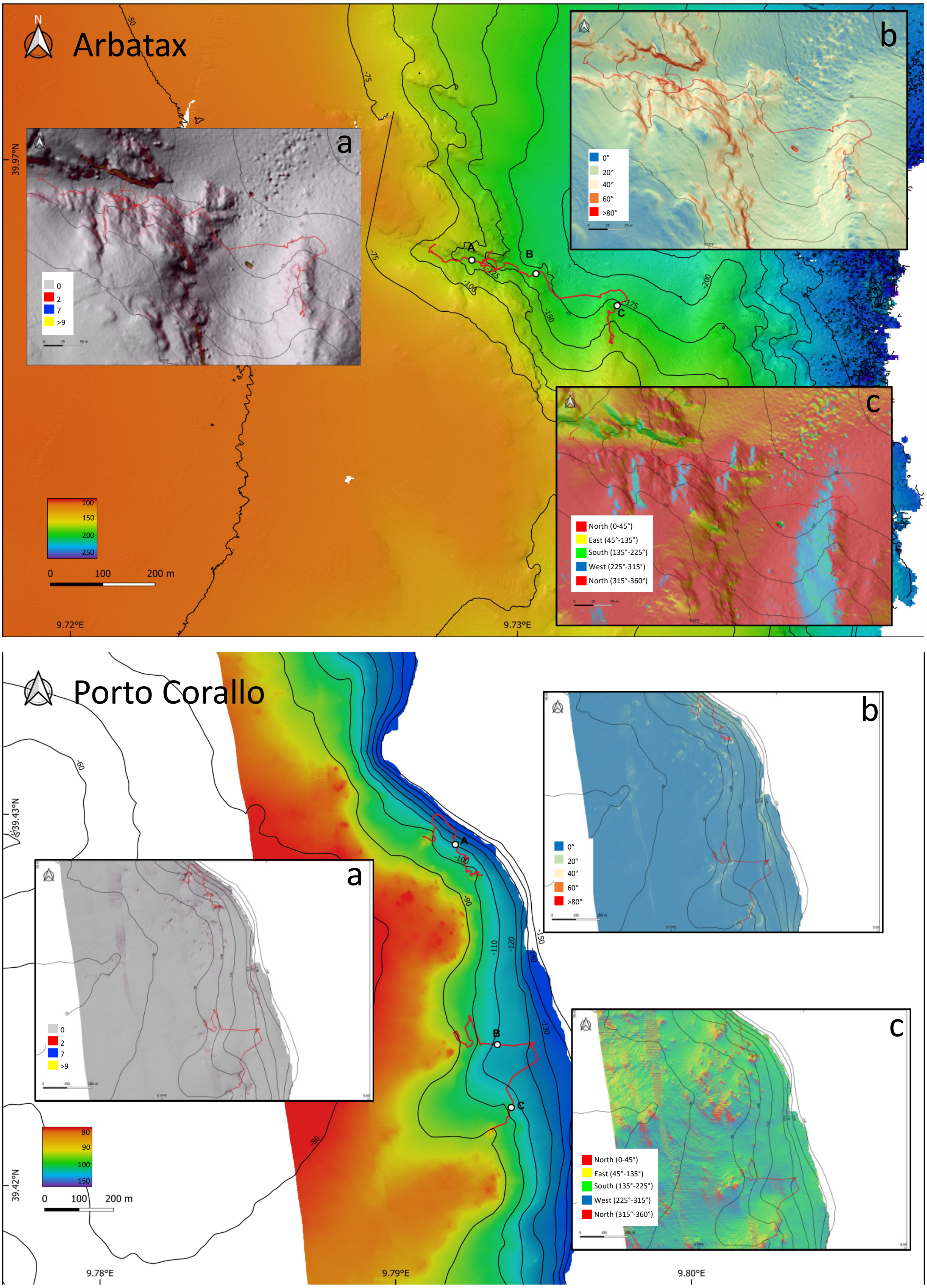

N
Arbatax
a
b
c
A
B
C
0
2
7
>9
0°
20°
40°
60°
>80°
North (0-45°)
East (45°-135°)
South (135°-225°)
West (225°-315°)
North (315°-360°)
100
150
200
250
0
100
200 m
-50
-75
-100
-125
-150
-175
-200
39.97°N
9.72°E
9.73°E
Porto Corallo
-60
-80
-90
-100
-110
-120
-130
-140
-150
80
90
100
150
39.43°N
39.42°N
9.78°E
9.79°E
9.80°E

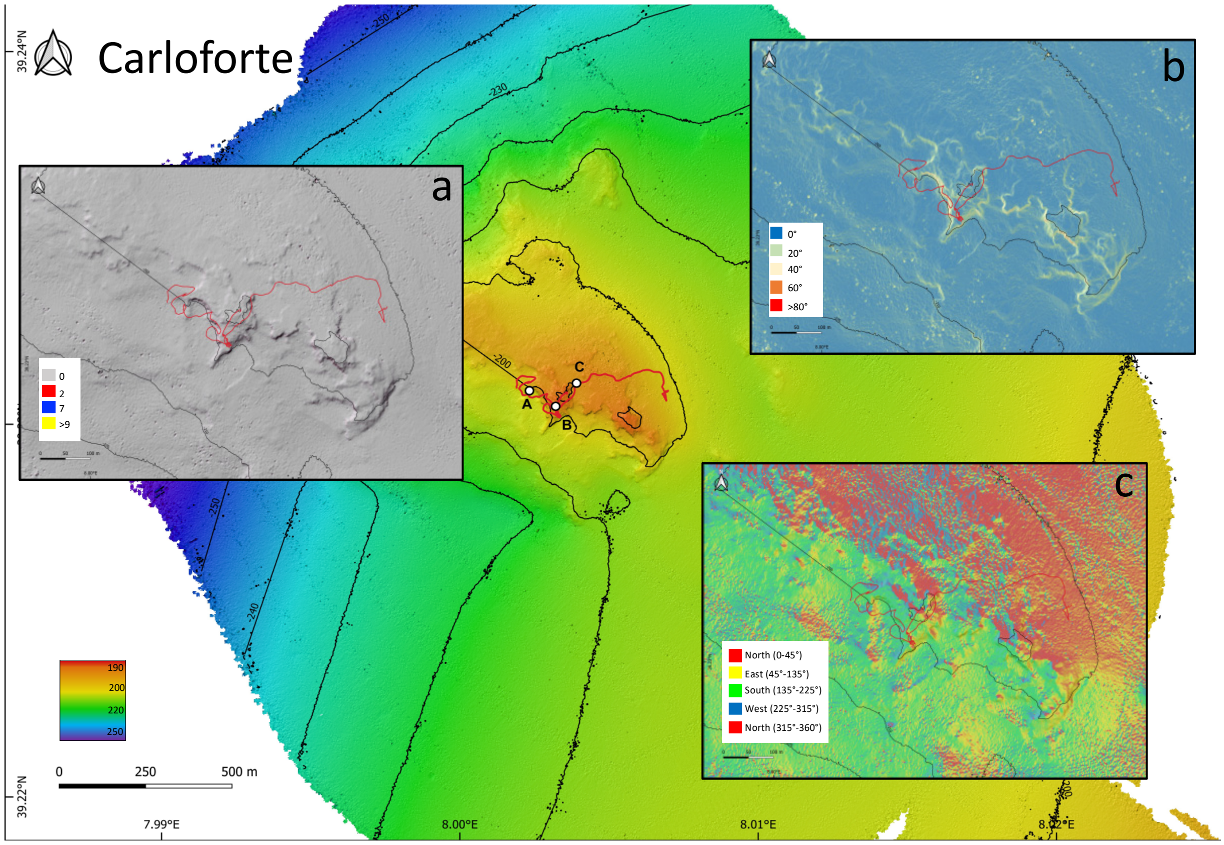

Figure S1. Bathymetric, TRI (a), Slope (b) and Aspect (c) map of the study areas from MBES data, showing the ROV navigation tracks (red lines), the sites ID and the approximate location of the 200-m long (A, B, C) transects extracted for the quantitative analyses and application of MACS index.

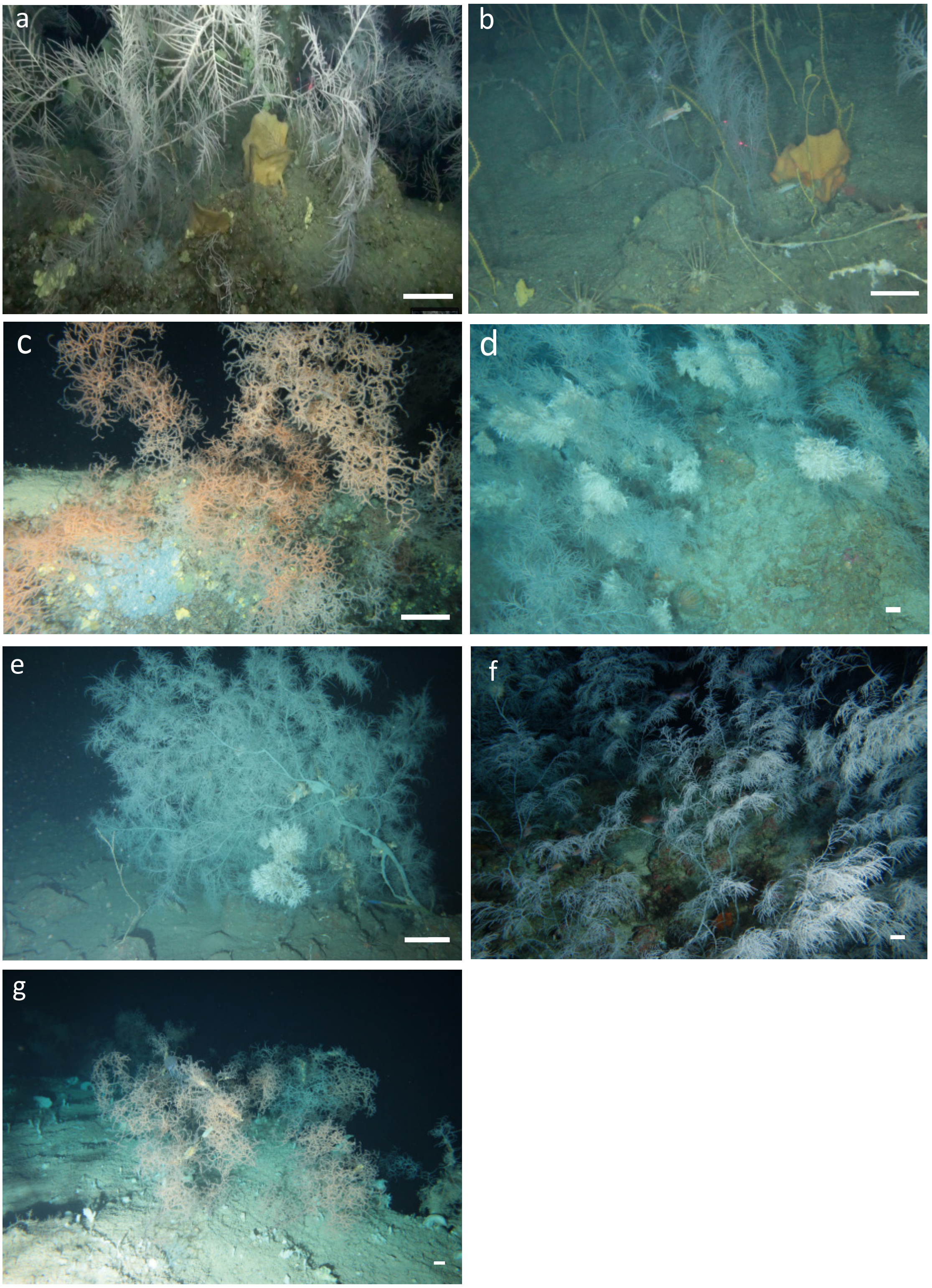

Figure S2. Selected ROV images showing black corals colonies at the seven investigated sites. (a) Colonies of *A. subpinnata* observed in Caprera site at 144m depth with colonies of *E. cavolinii* and *P. compressa* underneath the canopy; (b) colonies of *A. subpinnata* in Olbia site at 133m dwell with a dense facies of *V. flagellum*; (c) colonies of *L. glaberrima* on encrusted rocky boulder in Tavolara site at 177m depth; (d) colonies of *A. subpinnata* in Orosei site at 98m depth with several portions of the branches covered by *Salmicina-Filigrana implexa*; (e) big colony of *A. subpinnata* in Arbatax site at 140m depth with portions covered by the epibionts *Salmicina-Filigrana implexa* and other polychaetes species; (f) thick forest of *A. subpinnata* in Porto Corallo site at 95m depth with few individuals of *A. anthias* swimming underneath the canopy; (g) colonies of *L. glaberrima* in Carloforte site at 184m depth with capsules of *S. caniucla* hanging over the branches. Scale bar of approximately 10 cm.

Table S1*:* Geographic and geomorphological characteristics of ROV dives performed in the seven investigated sites. TRI: Terrain Ruggedness Index, TPI: Topographic Positioning Index.

Site	Transects code	Coordinates				Length	Depth	Average Slope	Average TRI	Average TPI	Prevalent Aspect
		Start		End		(m)	(m)	(°)			
Caprera	CA_T1	41.2902 N	9.6249 E	41.2902 N	9.6257 E	212	121-142	25	1.2	0.014	E
	CA_T2	41.3058 N	9.6335 E	41.3054 N	9.6334 E	256	127-144	27	1.3	0.01	S
	CA_T3	41.3054 N	9.6334 E	41.3048 N	9.6333 E	220	146-183	29	1.7	0.013	S
Olbia	OB_T1	41.0707 N	9.8028 E	41.0702 N	9.8011 E	205	126-147	47	0.9	0.57	E
	OB_T2	41.0701 N	9.8012 E	41.0681 N	9.8012 E	236	126-140	17	0.3	0.5	E
	OB_T3	41.0705 N	9.7981 E	41.0698 N	9.7973 E	221	100-120	37	0.9	-0.018	N
Tavolara	TV_T1	40.9119 N	9.9153 E	40.9107 N	9.9158 E	206	175-201	14	0.2	0.018	S
	TV_T2	40.9139 N	9.9002 E	40.9132 N	9.9014 E	261	107-140	27	0.5	0.13	E
	TV_T3	40.9195 N	9.9018 E	40.9186 N	9.9029 E	310	144-196	56	1.6	0.018	E
Orosei	OR_T1	40.3636 N	9.8942 E	40.3644 N	9.8941 E	265	95-101	21	1.3	0.58	W
	OR_T2	40.3646 N	9.8944 E	40.3658 N	9.8951 E	302	93-117	55	1.9	-0.035	W
	OR_T3	40.3628 N	9.8943 E	40.3635 N	9.8944 E	219	96-100	66	2.2	-0.11	S
Arbatax	AR_T1	39.9684 N	9.7282 E	39.9680 N	9.7293 E	237	108-143	40	0.8	0.017	N
	AR_T2	39.9681 N	9.7299 E	39.9675 N	9.7314 E	241	139-171	39	0.7	-0.033	N
	AR_T3	39.9675 N	9.7316 E	39.9668 N	9.7322 E	201	128-183	39	0.7	-0.046	N
Porto Corallo	PC_T1	39.4297 N	9.7918 E	39.4283 N	9.7929 E	240	90-126	25	1	-0.14	S
	PC_T2	39.4239 N	9.7922 E	39.4237 N	9.7942 E	301	82-111	17	2	0.06	W
	PC_T3	39.4238 N	9.7948 E	39.4214 N	9.7933 E	200	83-102	18	3.1	0.01	W
Carloforte	CR_T1	39.2312 N	8.0018 E	39.2310 N	8.0028 E	254	192-198	10	0.1	0.002	S
	CR_T2	39.2309 N	8.0027 E	39.2304 N	8.0031 E	210	188-195	16	0.2	0.02	S
	CR_T3	39.2303 N	8.0032 E	39.2316 N	8.0044 E	201	184-192	10	0.1	0.002	S

Table S2. A) Reference scores developed for the 12 components used in the MACS index. H, height; GL, general litter; LFG, lost fishing gears. B) Scores defining the environmental status classes for Is, Ii and MACS. Both tables are from Enrichetti et al. (2019).

A)

Components	Score 0	Score 1	Score 2	Score 3
**Index of Status (I_s)**				
Species richness (SR)	0	≤20	>20 ≤40	>40
Basal bio-cover (BC)	0	<1.5	≥1.5 <2.5	≥2.5
Coralline algea cover (CC)	0	<1.5	≥1.5 <2.5	≥2.5
Dominance of structuring species (DM)	SSD ≤ 0.5 org m^{-2}	1 sp.	2 sp.	≥3 spp.
Density of all strutcuring species (SSD)	SSD ≤ 0.5 org m^{-2}	≤2	>20 ≤5	>5
Mean height of dominant structuring species (SSH)	SSD ≤ 0.5 org m^{-2}	≤ 1/3 of 1/2 max H	> 1/3 ≤2/3 of 1/2 max H	> 2/3 of 1/2 max H
**Index of Impact (I_i)**				
Sedimentation (S)	0	<1.5	≥1.5 <2.5	≥2.5
Percent of entangled colonies (ENT)	0	≤20	>20 ≤40	>40
Percent of necrotic colonies (ENT)	0	≤20	>20 ≤40	>40
Percente of epibioneted colonies (EPB)	0	≤20	>20 ≤40	>40
Density of litter items (LD)	0	≤0.1	>0.1 ≤0.2	>0.2
Litter typology (LT)	Absence of litter	Presence of GL	Presence of LFG	GL+LFG

B)

Index of Status (Is)	Index of Impact (Ii)	MACS
≤ 35 = Bad	≤ 35 = Bad	≤ 35 = Bad
36-45 = Poor	36-45 = Poor	36-45 = Poor
46-55 = Moderate	46-55 = Moderate	46-55 = Moderate
56-65 = Good	56-65 = Good	56-65 = Good
≥ 66 = High	≥ 66 = High	≥ 66 = High

Table S3. List of species recorded within the investigated sites.

Phylum	Species	Caprera	Orosei	Arbatax	Olbia	Porto Corallo	Tavolara	Carloforte
Porifera	*Aplysina spp.*		+			+		
	Axinella spp.	+	+	+	+	+	+	
	Haliclona spp.							+
	Encrusting orange spongia			+	+			
	Encrusting white spongia			+	+			
	Encrusting blue spongia			+				
	Encrusting gray spongia					+		
	Erect brown spongia	+	+			+	+	+
	Erect white spongia	+	+		+	+	+	+
	Erect yellow spongia	+			+	+	+	+
	Erect orange spongia		+		+	+	+	
	Pachastrella monilifera	+		+	+	+		+
	Poecillastra compressa	+	+	+	+	+		
	Terpios gelatinosa	+						+
Cnidaria	*Hidrozoa spp.*	+	+		+	+	+	+
	Sertulariidae spp.	+	+	+		+	+	+
	Anthipates dichotoma			+	+		+	+
	Antipathes subpinnata	+	+	+	+	+	+	
	Caryophyllia spp.	+	+	+	+	+	+	+
	Cerianthus sp.		+			+	+	
	Dendrophyllia cornigera	+	+	+	+	+	+	
	Desmophyllum dianthus	+		+				+
	Leiopathes glaberrima						+	+
	Madrepora oculata						+	
	Parantipathes larix	+		+	+		+	+
	Savalia savaglia		+					
	Parazoanthus axinellae	+	+			+	+	+
	Acanthogorgia hirsuta	+				+	+	+
	Alcynium spp.	+	+	+	+		+	
	Bebryce mollis		+				+	+
	Callogorgia verticillata	+		+	+		+	+
	Corallium rubrum	+	+	+	+	+	+	
	Eunicella cavolini	+	+		+		+	
	Paramuricea clavata		+				+	
	Gerardia savaglia					+		
	Villogorgia bebrycoides	+					+	
	Viminella flagellum	+			+		+	
Annelida	*Serpulidae sp.*	+	+	+	+	+	+	+
	Salmacina-Filigrana implexa	+	+	+	+	+	+	

	Sabella sp.			+		+		
Tunicata	*Halocynthia papillosa*		+			+		
	Ascidia sp.					+	+	
Annelida	*Bonellia viridis*	+	+		+	+	+	+
Brachiopoda	*Gryphus vitreus*	+						
Mollusca	*Pseudosimnia carnea*					+		
	Gastropoda sp. 1						+	
	Gastropoda sp. 2					+	+	
	Nudibranchs sp.	+					+	+
	Peltodoris atromaculata		+			+		
	Ostrea sp.		+		+	+	+	
Arthropoda	*Plesionika sp.*					+		
	Crusyacea sp.						+	
	Munida sp.		+					
	Rochinia rissoana							+
	Pagurus sp.						+	
	Palinurus elephas		+		+	+	+	+
Bryozoa	*Reteporella grimaldii*	+	+				+	
	Myriapora truncata		+			+	+	
	Reteporella beaniana						+	
Echinodermata	*Echinaster sepositus*			+	+			
	Centrostephanus longispinus				+	+		
	Cidaridae sp.		+	+	+	+	+	
	Echinus melo	+	+		+		+	
	Holothuria sp. 1		+					
	Holothuria sp. 2		+	+	+	+	+	
	Hacelia attenuata	+	+			+	+	
	A. mediterranea	+	+		+		+	+
	Ophiothrix sp.	+					+	+
	Peltaster placenta	+	+	+	+	+	+	
Chordata	*Antias antias*			+	+	+		
	Benthecomete robustus						+	
	Scorpaena notata		+		+	+		
	Helycodenus dactylopterus				+			
	Murena elena	+					+	
	Galeus melastomus	+						
	Cerranus hepatus				+			
	Acantholabrus palloni	+		+				+
